# Hypoxia–ischemia is not an antecedent of most preterm brain damage: the illusion of validity

**DOI:** 10.1111/dmcn.13483

**Published:** 2017-06-28

**Authors:** Floyd Gilles, Pierre Gressens, Olaf Dammann, Alan Leviton

**Affiliations:** ^1^ Children's Hospital Los Angeles Los Angeles CA USA; ^2^ Inserm U1141 Hôpital Robert Debré Paris France; ^3^ Univ Paris Diderot Sorbonne Paris Cité UMRS 1141 Paris France; ^4^ Centre for the Developing Brain Division of Imaging Sciences and Biomedical Engineering KCL St. Thomas' Hospital London UK; ^5^ Tufts University School of Medicine Boston MA USA; ^6^ Hannover Medical School Hannover Germany; ^7^ Boston Children's Hospital and Harvard Medical School Boston MA USA

## Abstract

Brain injury in preterm newborn infants is often attributed to hypoxia–ischemia even when neither hypoxia nor ischemia is documented, and many causative speculations are based on the same assumption. We review human and animal study contributions with their strengths and limitations, and conclude that – despite all the work done in human fetal neuropathology and developmental models in animals – the evidence remains unconvincing that hypoxemia, in the fetus or newborn infant, contributes appreciably to any encephalopathy of prematurity. Giving an inappropriate causal name to a disorder potentially limits the options for change, should our understanding of the etiologies advance. The only observationally‐based title we think appropriate is ‘encephalopathy of prematurity’. Future pathophysiological research should probably include appropriately designed epidemiology studies, highly active developmental processes, infection and other inflammatory stimuli, the immature immune system, long chain fatty acids and their transporters, and growth (neurotrophic) factors.

**What this paper adds:**

- Fetal hypoxemia is rarely documented in brain injury studies.
- Animal studies fail to consider human–animal fetal anatomical differences.
- Putative treatments from animal models have not found clinical use.
- Observational studies constitute the only approach to etiological understanding.
- No convincing evidence yet that hypoxemia injures preterm brain. Encephalopathy of prematurity is preferable to hypoxia‐ischemia as a term for this disorder.
- Encephalopathy of prematurity is preferable to hypoxia‐ischemia as a term for this disorder.

## What this paper adds

- Fetal hypoxemia is rarely documented in brain injury studies.
- Animal studies fail to consider human–animal fetal anatomical differences.
- Putative treatments from animal models have not found clinical use.
- Observational studies constitute the only approach to etiological understanding.
- No convincing evidence yet that hypoxemia injures preterm brain. Encephalopathy of prematurity is preferable to hypoxia‐ischemia as a term for this disorder.
- Encephalopathy of prematurity is preferable to hypoxia‐ischemia as a term for this disorder.

This article is commented on by Paneth on page 115 of this issue.

This article's abstract has been translated into Spanish and Portuguese.

Follow the links from the abstract to view the translations.

## Resumen

***Hipoxia‐isquemia* no es un antecedente, en la mayoría de los casos de daño cerebral, en recién nacidos prematuros: la ilusión de la validez:**

El daño cerebral en los recién nacidos prematuros se atribuye frecuentemente a hipoxia‐isquemia incluso cuando ninguna de ellas ‐ hipoxia o isquemia ‐ están documentadas, y muchas especulaciones causales se basan en el mismo supuesto. En este estudio revisamos las contribuciones de los estudios realizados en humanos y en animales, con sus fortalezas y limitaciones, y concluimos que ‐ a pesar de todo el trabajo realizado en neuropatología fetal humana y en modelos de desarrollo en animales ‐ la evidencia sigue siendo poco convincente de que la hipoxemia, en el feto o en el recién nacido, contribuye significativamente a cualquier tipo de encefalopatía de la prematurez. Asignar un nombre causal inadecuado a un trastorno potencialmente limita las opciones de cambio en el futuro, considerando que nuestro conocimiento de las etiologías avanza. El único nombre, basado en la observación, que creemos apropiado es el de “*encefalopatía de la prematurez*”. La investigación fisiopatológica futura debería incluir estudios epidemiológicos diseñados apropiadamente para este periodo, considerando procesos de neuroplasticidad altamente activos, infección y otros estímulos inflamatorios, el sistema inmune inmaduro, ácidos grasos de cadena larga y sus transportadores, y factores de crecimiento neurotróficos.

## Resumo

**Isquemia‐hipóxica não precede a maior parte das lesões cerebrais em prematuros: a ilusão da validade:**

A lesão cerebral em lactentes prematuros é frequentemente atribuída a isquemia‐hipóxica mesmo quando nem a hipóxia nem a isquemia são documentadas, e muitas especulações causais são baseadas na mesma suposição. Nós revisamos contribuições de estudos humanos e em animais, com seus pontos fortes e suas limitações, e concluímos que – apesar de todo trabalho feito em neuropatologia fetal humana e desenvolvimento de modelos em animais – permanece inconclusiva a evidência que hipoxemia, no feto e no recém‐nascido, contribui significativamente para qualquer encefalopatia da prematuridade. Dar um nome causal inapropriado a uma desordem limita potencialmente nossa opção de mudança se o entendimento da etiologia avançar. O único nome baseado na observação que pensamos ser adequado é “encefalopatia da prematuridade”. Estudos patofisiológicos futuros deverão provavelmente incluir estudos epidemiológicos adequadamente desenhados, processos de desenvolvimento altamente ativos, infecção e outros estímulos inflamatórios, o sistema imune imaturo, ácidos graxos de cadeia longa e seus transportadores e fatores de crescimento (neurotróficos).

**Abbreviations**
BOOST: Benefits of Oxygen Saturation Targeting
COT: Canadian Oxygen Trial
SUPPORT: Surfactant, Positive Pressure, and Oxygenation Randomized Trial

## Introduction

### Disorders identified by an etiological name

Despite a complete lack of evidence, encephalopathy of prematurity has been attributed to hypoxia (ischemia). Physicians seem to rely on a limited number of heuristic principles, which reduce the complex tasks of assessing probabilities and predicting values to simpler judgmental operations. These heuristics sometimes lead to severe and systemic errors for which Tversky and Kahneman coined the term ‘The illusion of validity.’^1^ For instance, attributing a single cause to a disease should serve a purpose. Applying that attribution to the disease name, however, probably does not serve any useful purpose, as it is likely overly simplistic and potentially limits the options for change when our understanding of the etiologies advances.

To avoid errors associated with using an inappropriate causal label, some have suggested that the more general descriptive term ‘neonatal encephalopathy’ or ‘newborn encephalopathy’ should replace hypoxic–ischemic encephalopathy,^2^ or have used the term ‘encephalopathy of prematurity,’^3^ but have not turned away from the original term ‘hypoxic–ischemic encephalopathy.’^4^ Given the term ‘hypoxic–ischemic encephalopathy’, we are not surprised when most neonatal animal studies use some form of asphyxia. We are not denying that infants born very‐low‐birthweight and preterm are at risk of multiple neurological dysfunctions, multiple abnormalities on imaging, or multiple abnormalities at autopsy. We are suggesting that while hypoxia may be behind some abnormalities, most are the result of multiple contributions and consequently experimental work be directed toward other etiologies as well.

In this review, we focus on the encephalopathies seen in newborn infants born preterm, lesions attributed to hypoxia or hypoxia–ischemia, animal studies with their strengths and limitations, and conclude with the design of an ideal epidemiological study.

### Why is brain injury so much more common in preterm newborn infants than in those born near term?

The association between very preterm birth and subsequent neurological deficit is attributable to seven factors and while these vulnerabilities may interact with hypoxia and they may also interact with many other antecedents. First, some immature brain vulnerability can be attributed to highly active developmental processes such as dendritic or axonal growth (particularly growth cone proliferation), vasculogenesis, myelinogenesis, and angiogenesis.^5^ Second, a paucity of essential long chain fatty acids^6^ or appropriate fatty acid transporters such as Mfsd2a can further increase vulnerability. Third, newborn infants born very preterm appear to be unable to synthesize some growth factors in the amounts needed for normal development.^7^ Fourth, a low supply of such growth factors may be inadequate to protect against adversity.^8^, ^9^ Fifth, the infant born preterm is exposed to a host of potentially harmful exposures before, during, and after delivery, with many differing from those experienced by infants born close to term.^5^ Sixth, immature immune system stimulation potentially results in an overly intense inflammatory response that is likely sustained for some time.^10^ Seventh, inflammation diminishes the blood–brain barrier of newborn infants born very preterm.^11^

## Hypoxia–Ischemia

The term ‘hypoxia**–**ischemia’ conflates two very different physiologies, and we choose to separate them.

### Hypoxia

Hypoxia, or more appropriately hypoxemia, if sufficiently prolonged induces energy failure**,** and is associated with loss of neuronal function, much less in neonatal than in adult animals, and least in animals born preterm.^12^ The very preterm brain normally has much lower aerobic requirements than the brain at term.^13^ But, to the best of our knowledge, hypoxia alone, with sustained cerebral perfusion, has not been shown to cause brain lesions, either in preterm, term, or adult human or in experimental animal studies. While certain selective neuronal losses in adult brain have been attributed to hypoxia such specificity has yet to be verified.

### Ischemia

Ischemia, a general or focal restriction in tissue blood supply, also induces energy failure, but is far more complex than hypoxia in that it also diminishes blood component availability, and limits brain metabolic waste removal. Often this is due to systemic hypotension or cerebral vascular occlusion. Experimentally, hypotension is very important in producing lesions after umbilical artery occlusion.^14^, ^15^ Further, hypocapnia seems to increase the risk the risk of cerebral palsy (CP),^16^ presumably by attenuating local cerebral vascular supply. In newborn infants born preterm, however, hypotension does not appear to account for any brain ultrasound abnormality within the first 10 days,^17^ but this ‘negative finding’ has limited value because ultrasound scans do not provide an adequate view of cortical arterial borderzone regions.

## Human Studies

Because even mild hyperoxia appears to increase the risk of retinopathy of prematurity, neonatologists want to minimize the occurrence of hyperoxia, while at the same time avoiding levels of hypoxia they think might injure the brain. Three large, multicenter clinical trials were organized in an effort to find the optimum pulse oximeter oxygen saturation (SpO_2_) target ranges for infants born extremely preterm.^18^, ^19^, ^20^ All three compared ‘restricted’ oxygen exposure (defined as an oxygen saturation [SpO_2_] in the 85%–89% range) to liberal exposure (SpO_2_, 91%–95%) among infants born extremely preterm (<28wks’ gestation at birth).

The Surfactant, Positive Pressure, and Oxygenation Randomized Trial (SUPPORT) was conducted in the United States (*n*=1316)^18^ while the Canadian Oxygen Trial (COT) was a multinational trial enrolling newborn infants in both Canada and the United States (*n*=1201).^19^ The Benefits of Oxygen Saturation Targeting II (BOOST II) trials included three trials conducted in the United Kingdom (*n*=973), Australia (*n*=1135), and New Zealand (*n*=340).^20^ After publication of the first results from the SUPPORT trial,^18^ the data monitoring committees of the similar BOOST II trials in Australia and the United Kingdom terminated recruitment early but the children enrolled in these studies continued to be followed. This allowed the results from the Australian and United Kingdom components to be combined with results of the New Zealand component (of the BOOST II trial), which had already completed recruitment.

All three studies, the SUPPORT,^18^ BOOST II trials (New Zealand component only [BOOST‐NZ]),^20^ and COT,^19^ assessed death or disability before postnatal age 18 to 24 months as a primary outcome. In none of the trials was the risk of death or disability significantly elevated (SUPPORT: risk ratio=1.1; 0.9–1.3; COT: risk ratio=1.04; 0.9–1.2; BOOST‐NZ: risk ratio=0.9; 0.7–1.1). Combining all three studies showed neither increased nor decreased risk of death or disability by age 18 to 24 months (risk ratio=1.02; 0.9–1.1).^21^

‘Death or disability’ as the primary outcome is difficult for those of us who do not favor composites of (vastly) different endpoints. This composite is often justified because death and disability are seen as competing risks: you have to survive to be at risk of disability. So why not first evaluate the risk of death, and then evaluate among survivors the risk of indicators of brain injury?

The COT report did not include death alone as an individual outcome, but the SUPPORT and BOOST trials did. Although neither the SUPPORT nor the BOOST trials alone found that children with restricted oxygen exposure were at increased risk of early death, combining data from these two trials allowed the increased risk of death before discharge associated with restricted oxygen exposure to achieve statistical significance (risk ratio=1.2; 95% confidence interval: 1.03–1.4).^21^

Although none of the three studies had an indicator of impaired neurodevelopment as a primary outcome, all included a Gross Motor Function Classification System (GMFCS) level of II or higher as a secondary outcome. The GMFCS was developed to classify the motor function of children who have a diagnosis of CP. It was not intended for use among children who are not thought to have CP. GMFCS level I is defined as able to walk indoors and outdoors and climb stairs without using hands for support, and able to run and jump. The earlier in the 18‐ to 24‐month age range, the greater the likelihood the child will be identified as having a GMFCS level at or above II, regardless of whether or not the child will develop cerebral palsy. Indeed, the developers of the GMFCS who assessed children at a mean age of 19 months suggested that ‘there is a need for reclassification at age 2 or older as more clinical information becomes available.^22^

Consequently, the likelihood of misclassification deserves consideration as we assess the quality of these studies. Indeed, the authors of the meta‐analysis concluded, ‘Using the Grades of Recommendation, Assessment, Development, and Evaluation (GRADE) criteria, we found that the quality of evidence for these outcomes (including neurodevelopmental outcomes) was moderate to low.^21^

With this caveat, the risk of a GMFCS level at or above II was not significantly elevated in the SUPPORT trial (risk ratio=1.2; 0.7–2.1), the COT trial (risk ratio=0.97; 0.6–1.67), or the BOOST‐NZ trial (risk ratio=0.7; 0.2–2.2). In a meta‐analysis combining all three studies, the risk ratio was very close to 1.0 (risk ratio=1.03; 0.7–1.5).^21^ Thus, we can say, given the limited evidence available, that hypoxia at levels clinicians can tolerate does not appear to increase the risk of motor limitation at 18 to 24 months. This statement is especially important in light of evidence that the restricted levels of oxygen appear to increase the risk of death. Death? Yes. Disability? No.

The only observational study that evaluated the hypothesis that oxygen lack is an antecedent of white matter injury, CP, low Bayley Scales, and microcephaly in newborn infants born extremely preterm found that every one of the five blood gas derangements during the first three postnatal days (hypoxemia, hyperoxemia, hypocapnia, hypercapnia, and acidemia) was associated with multiple indicators of brain injury.^23^ The patterns of associations varied considerably and not necessarily in a cohesive manner. Rather than inferring that the blood gas derangements caused the brain injury, a more plausible interpretation is that the multiple derangements are indicators of immaturity/vulnerability and illness severity.

In light of these reports, we draw the inference that no documentation has yet been provided that hypoxia contributes to brain injury in very preterm newborn infants, even when such levels of hypoxia appear to increase the risk of death.

## Fetus

In this day of evidence‐based medicine, one expects that the clinical diagnosis of ‘hypoxic encephalopathy’ implies that there has been a documented sufficiently long and severe hypoxic exposure. However, the entity in newborn infants born at term labeled ‘hypoxic–ischemic encephalopathy’ often does not follow hypoxic–ischemic exposures.^24^, ^25^

Moreover, the embryo and fetus develop in a markedly hypoxic environment,^26^ with oxygen concentrations of 0.076 to 7.6 mmHg compared to those in adults of 11.4 to 53.2 mmHg,^27^ and local hypoxic regions exist within embryonic and fetal brain, heart, and mesenchyme, apparently promoting embryonic and fetal vascularization and organogenesis.^28^

Blood pressure and flow relationships in newborn infants born preterm differ markedly from those in adults. Three fetal circulation shunts, ductus venosus, foramen ovale, and ductus arteriosus, permit oxygenated fetal blood to bypass liver and lungs and enter the left heart, but still only half the blood is oxygenated during one fetal circulation cycle. Consequently, adult concepts cannot be automatically applied to the preterm fetus. In fetal lambs, arterial pressure is low and systemic arterial blood gas tensions are asphyxial by adult standards. Cerebral vascular resistance declines between mid‐gestation and term.^29^

## Lesions Attributed to Hypoxia‐Ischemia

Neuronal death – regardless of where along the axis from apoptosis through necrosis, necroptosis,^30^, ^31^ and pyroptosis – by itself gives no clue about etiology, except perhaps when occurring in borderzone regions, in part because neuronal death results from many different metabolic, vascular, or inflammatory derangements, each often the consequence of multiple different antecedents.

The numerous morphological lesions attributed to hypoxia‐ischemia over the last 60 years include the following: hemorrhage in many brain locations; edema; endothelial injury; necroses in many locations including gray matter and white matter; infarct; white matter gliosis; atrophies; cysts in many locations; myelination delay; ventriculomegaly; hydranencephaly; and finally, malformation.^32^, ^33^, ^34^ Thus, the term hypoxic–ischemic encephalopathy fails to predict specific structural outcomes. The isolated occurrence of each entity can be attributed to the same etiology only if other phenomena (e.g. specific time and specific location) are also important; if they are, then the entity is really multifactorial.

## Animal Models are Needed to Understand Preterm Human Brain Abnormalities

Good translational research requires suitable animal models, yet interspecies differences limit the relevance of models mimicking human pathophysiology. In fact, some students of animal models are not sure how well animal models in general match human cancer, cerebral infarcts, stroke therapies, microglia, and complex human behavioral disorders. Common deficits have been poor methodological quality,^35^ low statistical power, poor statistical analyses, and lack of blinding and randomization. Investigators of developmental brain injury have focused on cerebral maturation, blood flow, metabolism, and white matter injury, but other areas may need attention. In addition, some animal models do not resemble the human pathological abnormality to provide therapeutic candidates. For instance, a model producing any brain injury is not a model of human focal white matter necrosis.^36^ The ideal model replicates all aspects of the human disease being modeled, including exposures, dysfunctions, and morphology. Because this is rarely practicable, close approximations are acceptable with caveats, as long as we acknowledge their limitations. For instance, if the effect of brain hypoxia is being modeled, then cerebral blood flow must be held constant and the resultant brain injury must resemble the specific brain abnormality modeled.

### Developmental differences between humans and other mammals

Timing, opportunity window, ischemia duration, drug dose, species, sex differences, comparable fetal developmental age, and underlying diseases all need consideration when designing and evaluating models of fetal cerebral abnormalities.^37^ Unfortunately some of these characteristics have been ignored. Human infants are advanced at birth relative to rat and rabbit. Thus rat at P1 or P2 is roughly equivalent to human at 0.45 of its fetal developmental time and rat at P7 is roughly equivalent to human at 0.60 gestation. Similarly, rabbit at E22 or E25 is roughly equivalent to human at 0.35 gestation, at least based on tract appearance.^38^ Still, these models offer opportunities to study effects of various agents or procedures on fetal brain.

### Cerebral autoregulation in preterm fetuses

Term and preterm animal fetuses respond to asphyxia (the combination of hypoxia, hypercapnia, and metabolic acidosis) in a qualitatively similar manner.^13^ Sometimes the asphyxia does not occur in isolation. For example, umbilical cord occlusion decreases brain oxygen availability, but it also reduces fetal systemic blood pressure, diminishes blood component availability, and limits brain metabolic waste removal. Animal studies suggest that hypotension timing during severe asphyxia influences location and magnitude of cerebral injury, likely because of the close relationship between fetal blood pressure maintenance and changes in brain perfusion. Nevertheless, the concept of cerebral autoregulation in preterm humans continues to evolve.

### Anatomical differences between human and other mammalian fetuses

Animal and human fetal differences need consideration when designing studies. For instance, the popular sheep fetus model has a different anatomy than the human fetus. The sheep fetus has a longer intrathoracic inferior vena cava, different liver position, two umbilical veins, higher body temperature, lower hemoglobin, shorter pregnancy, and a syndesmochorial cotyledonary placenta rather than the human hemomonochorial placenta, all potentially important in an experimental study. Further, unlike the human fetus,^39^ the fetal sheep's major intrauterine brain growth spurt occurs well before birth, resulting in much earlier cerebral myelination,^40^ and a much smaller brain. Sheep also have a different cerebral blood supply with an interposed carotid rete mirabile.^41^ This point is crucial because the fetal lamb does not have a proximal internal carotid segment. An external carotid branch supplies the rete (composed of a bed of fine branches) interposed between systemic and cerebral circulations, raising questions of whether sheep fetal cerebral circulation is sufficiently similar to human fetal circulation to be a model of human fetal brain circulatory abnormalities. Similar questions about the relevance to human disease need consideration when interpreting results of rodent models.

## Selected Animal Studies

The following remarks are offered as commentary to accompany a reading of Table SI (online supporting information).

### Baboons

The two baboon studies (Inder et al.^42^ and Loeliger et al.^43^) were chosen to indicate that preterm birth, alone without additional manipulation, is not benign and, in these primates, results in cerebral injury (Table SI).

The remaining 10 models of acquired human prenatal cerebral abnormality attributed to hypoxia in Table SI were chosen because of their strengths and the care with which they were executed. The researchers who used these models considered the animal developmental stages equivalent to preterm human infants, although this may be inaccurate.^38^ In addition to hypoxia, these models required occlusion of carotid, umbilical, maternal descending aorta, or uterine arteries, thus potentially lowering cerebral blood flow. Blinded observers were utilized in five studies. Only one used randomization, despite the criteria for human clinical studies requiring randomization and double blinding, potentially introducing bias.^44^ In addition, failure to uniformly and effectively monitor the animal's response to anesthesia, particularly blood pressure, poses inferential problems. Fetal brain hypoxia degree was not evaluated, although systemic PaO_**2**_ showed a 30 to 40 percent decrease in two studies.^44^, ^45^

### Fetal sheep

We included the first sheep model (Wassink et al.^46^) because it indicates that fetal umbilical artery occlusion causes bradycardia, transient hypertension, and then hypotension, less in younger than in more mature fetuses, but brains were not examined. Keunen et al.'s study indicates that fetal sheep at midgestation do not get neuronal injury with 10 to 20 minutes of umbilical artery occlusion,^44^ but Mallard et al. found that fetal sheep later in gestation did get neuronal injury with daily microsphere injection into the umbilical circulation.^47^ George et al.'s study, furthermore, found basal ganglia, thalamic, hippocampal, and medullary injury after umbilical cord occlusions at roughly midgestation.^48^ Differences among these studies probably reflect not only fetal age, but also differences in study design. Brain energy supplies were not monitored, nor was fetal brain hypoxia tolerance measured.

### Rodents

The three postnatal rat studies (Rice et al.,^49^ McQuillen et al.,^50^ and Sizonenko et al.^51^) found cortical and basal ganglia injury with white matter necrosis, subplate neuronal loss, or white matter loss after carotid occlusion and hypoxia. The Buser et al., Drobyshevsky et al., and Derrick et al. rabbit studies, all done at very early developmental stages, found gray and white matter injury after maternal descending aorta occlusion.^45^, ^52^, ^53^

### Models requiring circulatory impairment

In the absence of evidence in human newborn infants born very preterm that hypotension causes brain injury,^17^ the models requiring circulatory impairment cannot be viewed as suitable models of the preterm human fetus. Global cerebral ischemia secondary to maternal or fetal vascular occlusion is hardly appropriate, given what we know about the preterm human fetus. We have included fetal mortality rates, when reported, to show that for several of these experiments investigators were working close to fetal lethality. Importantly, no model suggested testable therapeutic interventions.

## Synthesis and Outlook

Hypoxia beyond the normal fetal hypoxic environment as an antecedent has not been documented as causing injury in the preterm fetus. The fetus has a different blood supply as blood pressure and flow relationships differ from those in the adult. The large number of pathological conditions attributed to hypoxia indicates that the term ‘hypoxic encephalopathy’ has limited predictive power. Experimental animal studies have failed to take anatomical differences into consideration, leave much to be desired, and have not resulted in therapeutic candidates.

Despite all studies in animals and humans, the evidence remains unconvincing that hypoxemia, in the fetus or newborn infant, contributes appreciably to any encephalopathy of prematurity. Thus, the criteria for attributing perinatal brain injury to hypoxemia have not been met.

We recommend that until evidence becomes available, hypoxia–ischemia should not be viewed as contributing to the occurrence of what is included under the umbrella of ‘encephalopathy of prematurity’.

## Supporting information


**Table SI**: Key characteristics of several animal models
