# Supplementary material for: Hypoxia–ischemia is not an antecedent of most preterm brain damage: the illusion of validity
Source: Dev Med Child Neurol. 2017 Jun 28;60(2):120–5. doi: 10.1111/dmcn.13483 (PMC5745320; doi:10.1111/dmcn.13483)
Supplement: Supplementary file 1 — Table SI: Key characteristics of several animal models [file DMCN-60-120-s001.docx]

| **Table SI**: Key characteristics of several animal models | | | | | | | | | |
| --- | --- | --- | --- | --- | --- | --- | --- | --- | --- |
| **Author** | **Human preterm brain pathologic abnormality modeled** | **Animal** | **How was ‘hypoxia’ produced?** | **How were control animals manipulated?** | **How hypoxic was fetal brain?** | **How long did hypoxia/ ischemia last?** | **Was cerebral circulation concomitantly impaired and how monitored?** | **Resulting brain damage details** |  |
| Inder et al.^42^ | Any abnormality | Baboon (Papio papio) E125 or 0.6 its gestation (*n*=28) | Preterm delivery only. With standard neonatal intensive care for 14 days | Term infants (*n*=?)  Blinded observers  Point counting | No hypoxia/  ischemia | No hypoxia/  ischemia | Systemic blood pressure heart rate, O2 saturation, ekg, electrolytes, and blood counts monitored  Hypotension managed. No details | Brain weight reduced in all preterm animals  White matter damage 50%, hemorrhage in multiple locations, neuron loss in hippocampus (some animals), basal ganglia 5% and cortex 25%  Comment: no mortality rate |  |
| Loeliger et al.^43^ | Effects of ventilator therapies on preterm delivered animals | Baboon E125 or 0.6 its gestation | Preterm delivery with early (*n*=6) or late (*n*=5) positive pressure airway | Gestational controls (*n*=4)  Blinded observers | No hypoxia/  ischemia | No hypoxia/  ischemia | No hypoxia/  ischemia | Brain and body weights, cerebral volume, and surface folding index reduced in both experimental groups. Early continuous positive airway pressure associated with less cerebral injury  Comment: small numbers of animals  No mortality rate |  |
| Wassink et al.^46^ | Hemodynamic responses to severe asphyxia | Sheep at 0.6 (*n*=12), 0.7 (*n*=12), and 0.85 (*b*=8) gestation | Fetal umbilical artery occlusion | None  Probably not needed | Not study point | 15–30 minutes complete umbilical cord occlusion | Not study point | Bradycardia and hypertension followed by hypotension. Less in younger fetuses. EEG suppressed |  |
| Keunen et al.^44^ | Neuronal damage in immature fetuses depends on duration of umbilical cord occlusion | Fetal sheep E85–90 (midgestation) | Umbilical artery occlusion 10, 15, or 20 minutes (total *n*=32) | Sham fetuses similar instrumentation, but no occlusion | Fetal systemic pressure, blood gasses, pH, arterial O2 monitored | 10–20 minutes | Not measured | Bradycardia  No neuronal damage in isocortex, hippocampus, or cerebellum despite severe acidosis, hypercapnia, and hypoxia. Comment: no mortality |  |
| Mallard et al.^47^ | Chronic fetal hypoxemia | Fetal sheep E120–140 | Daily injections of microspheres into umbilical  Circulation from E120–E140 | Sham 5 control fetuses no microspheres | Fetal systemic PaO2 decreased ~30–40% | E120–E140 | Not measured | Cortical gliosis, reduced myelination. Increased diameter of brain capillaries, decreased P cells |  |
| George et al.^48^ | Assess fetal heart rate and EEG activity after asphyxia | Sheep E91 0.6 gestation (*n*=27) | Umbilical cord occlusion, 20 (*n*=7) or 30 minute (*n*=10) | Sham occlusions (*n*=10) randomly assigned | Fetal systemic arterial samples | 20 or 30 minutes | Not measured | Bradycardia. Seizures,  striatal, thalamic, hippocampal, and medullary damage |  |
| Rice et al.^49^ | Any brain damage  Post hoc | Rat Postnatal day 7 | Carotid occlusion plus hypoxia (8% O2 3.5 hours)(*n*=25) | Carotid ligation or hypoxia or neither.  Blinded observer  Controls (*n*=24) | Not measured | 3.5 hours | Not measured | Gray matter cortex striatum and hippocampus; white matter necrosis. 100% mortality at 7.5 minutes of total anoxia |  |
| McQuillen et al.^50^ | Subplate neurons  Post hoc | Rat P1 or P2 | Carotid Occlusion plus 5.6% O2 (*n*=64) | Sham (*n*=?) | Not measured | 3.0 hours | Not measured | Subplate neurons maximally vulnerable; also cells in intermediate and subventricular zones as well as thalamus. P1 mortality 31%; P2 mortality 19%. Skin temp maintained |  |
| Sizonenko et al.^51^ | Diffuse white matter injury | Rat, 3 day old | Carotid occlusion + 6% O2 30 min (*n*=25); carotid ligation (*n*=8) | Sham (*n*=5).  No intervention | Not measured | 30 minutes | Not measured | Unilateral reduction in hemispheral white matter and parietal cortex; no thalamic, striatal, hippocampal reduction. Mortality 2 HI group; 0 in other 2 groups |  |
| Buser et al.^45^ | White matter injury | Rabbit. E22 (*n*=48 survival until E23). E22 (*n*=7 survival E29) E25 (*n*=11 survival until E29) | Maternal descending aorta occlusion (called hypoxia) | Controls not manipulated (*n*=23 at E23) (*n*=16 at E26) (*n*=16 at E29).  Blinded observers | Immediate bradycardia | 40 minutes | Drop in cerebral microvascular blood flow | Oligodendroglial lineage cells counted. White matter resistant at E22 but not at E24 or E25  Gray matter damage in cortex, basal ganglia, and thalamus HI animals |  |
| Drobyshevsky et al.^52^ | White matter injury responsible for hypertonia  Diffusion tensor imaging study | Rabbit E22 (0.7 gestation). Hypertonic kits (*n*=32) compared to nonhypertonic kits (*n*=20) and to controls (*n*=19) | Maternal descending aorta occlusion (called hypoxia) | No sham  Masked observer | Not done | 40 minutes | Not measured | White matter damage in 80% of fetuses, but did not indicate why the remaining failed to get lesions. Gray matter damage particularly with ventriculomegaly  Comment: mortality of hypertonic kits at P5 47% |  |
| Derrick et al.^53^ | Model placental insufficiency | Rabbit E21 or E22 | Maternal descending aorta occlusion 30 (*n*=26), 37, 38, or 40 (*n*=102 HI E22 *n*=61 at E21) minutes. Controls (*n*=129) | Controls were done; manipulation not mentioned | Not measured. Immediate bradycardia | 40 minutes | Not measured. Systemic blood gases monitored after H/I | Hypertonia in 30% of H/I 40 minutes  In 5 controls and 9 exp pups increased microglia in cortex, corona radiata, caudate-putamen, ventral thalamus, and hippocampal formation. Comment: mortality rate 52% after 40 minutes H/I |  |
